# A 90-Day Toxicology Study of Meat from Genetically Modified Sheep Overexpressing *TLR4* in Sprague-Dawley Rats

**DOI:** 10.1371/journal.pone.0121636

**Published:** 2015-04-13

**Authors:** Hai Bai, Zhixian Wang, Rui Hu, Tongtong Kan, Yan Li, Xiaosheng Zhang, Jinlong Zhang, Ling Lian, Hongbing Han, Zhengxing Lian

**Affiliations:** 1 Beijing Key Laboratory of Animal Genetic Improvement, China Agricultural University, Beijing, P. R. China; 2 School of Life Science, Shanxi Datong University, Datong, Shanxi, P. R. China; 3 Tianjin Institute of Animal Sciences, Tianjin, P. R. China; University of Lleida, SPAIN

## Abstract

Genetic modification offers alternative strategies to traditional animal breeding. However, the food safety of genetically modified (GM) animals has attracted increasing levels of concern. In this study, we produced GM sheep overexpressing *TLR4*, and the transgene-positive offsprings (F1) were confirmed using the polymerase chain reaction (PCR) and Southern blot. The expression of *TLR4* was 2.5-fold compared with that of the wild-type (WT) sheep samples. During the 90-day safety study, Sprague-Dawley rats were fed with three different dietary concentrations (3.75%, 7.5%, and 15% wt/wt) of GM sheep meat, WT sheep meat or a commercial diet (CD). Blood samples from the rats were collected and analyzed for hematological and biochemical parameters, and then compared with hematological and biochemical reference ranges. Despite a few significant differences among the three groups in some parameters, all other values remained within the normal reference intervals and thus were not considered to be affected by the treatment. No adverse diet-related differences in body weights or relative organ weights were observed. Furthermore, no differences were observed in the gross necropsy findings or microscopic pathology of the rats whose diets contained the GM sheep meat compared with rats whose diets contained the WT sheep meat. Therefore, the present 90-day rat feeding study suggested that the meat of GM sheep overexpressing *TLR4* had no adverse effect on Sprague-Dawley rats in comparison with WT sheep meat. These results provide valuable information regarding the safety assessment of meat derived from GM animals.

## Introduction

The first genetically modified (GM) animal was reported in 1985 [1]. Since then, transgenic techniques have been applied to produce GM animals with varying degrees of success. GM sheep expressing high levels of circulating growth hormones were produced by microinjection of ovine metallothionein-ovine growth hormone fusion genes [2]. Subsequently, the insulin-like growth factor 1 and *BChE* gene were transferred to the sheep to improve their wool production and to protect against chemical agents, respectively [3, 4]. In addition to improving farm animal productivity and producing foreign protein as GM animal bioreactors, these transgenic techniques can be used to improve the health status and disease resistance of the animals more widely. For example, GM chickens expressing a short-hairpin RNA suppressed avian influenza transmission [5], *PrP* ^*C*^-deficient (*PRNP* ^*−/−*^) cattle were resistant to prions (BSE, bovine spongiform encephalopathy) [6], and GM cows that secreted lysostaphin resisted *S*. *aureus*-induced *mastitis* [7]. Furthermore, GM sheep that expressed the *visna virus* envelope gene could be genetically resistant to infection from lentiviral pathogens (*visna virus*) [8].

The toll-like receptor (TLR) family, which is one of the pattern recognition receptors (PRRs), plays an important role in innate immunity. Until now, at least 13 members have been found to belong to this family. Among them, TLR4 protein can recognize lipopolysaccharide (LPS), which is the principal component of the outer membrane of gram-negative bacteria [9, 10]. Under the stimulus of LPS, TLR4 can activate nuclear factor-kappa B (NF-κB) by MyD88-dependent or MyD88-independent signal pathways, a process which mediates the activation of proinflammatory cytokines and type 1 interferon genes. Thus, this particular activation plays a key role in regulating inflammatory and immune responses [11–13].

Some gram-negative bacteria, such as *Escherichia coli*, *Pasteurella*, *Salmonella* and *Brucella*, can infect many mammal species, including sheep, goat, cattle, buffalo, pig, mouse and human. Given the many species that could be put at risk by infection from these bacteria, there is a significant importance on the safety of food from the animal industry, especially in regards to human health. It has been reported that the overexpression of *TLR4* resulted in a survival advantage in GM mice during a greater percentage of *Salmonella* infection [14]. In our previous studies, we successfully cloned the sheep *TLR4* gene and produced GM sheep through microinjection. These GM sheep had increased release of inflammatory cytokines, initiated inflammatory responses rapidly and enhanced innate immunity significantly after LPS stimulation. The results indicated the potential for breeding sheep with disease resistance [15, 16].

Although transgenic techniques offer considerable advantages in animal breeding, GM animal food still represents a challenge in terms of food safety assessment. Accordingly, research investigations on the safety of food products derived from GM food and feed have been continuously performed. In 2008, a document “Guideline for Conduct of Food Safety Assessment of Foods Derived from Recombinant-DNA Animals” (CAC/GL 68–2008) was issued. Similar regulations were drawn in the European Union [17] and China [18].

The present study is part of a Chinese research project entitled “National Transgenic Creature Breeding Grand Projects”. The project is designed to develop scientific methodologies for assessing the safety of GM sheep meat. The objective of the 90-day feeding study in Sprague-Dawley rats was to evaluate the safety of meat from GM sheep overexpressing *TLR4* in an animal model, specifically when compared with wild-type (WT) sheep meat. Prior to the 90-day feeding study, a sample each of minced meat from GM sheep and WT sheep were subjected to a comprehensive analysis as the compositional data could provide the general information necessary to explain any possible effects that would be observed in our study. This study may provide a scientific base for the further biosafety assessment of foods derived from GM animals and offer valuable information for the future safety assessment of GM food.

## Materials and Methods

### Ethics statement

All experimental animal protocols were approved and performed in accordance with the requirements of the Animal Care and Use Committee at China Agricultural University (approval ID 2013–011). All surgeries were performed under sodium pentobarbital anesthesia, and all efforts were made to minimize any suffering experienced by the animals used in this study.

### Production and screening of GM sheep

*TLR4* gene (GU461886) was derived from sheep (*Ovis aries*) and cloned by our laboratory in 2011. Genetically modified sheep were produced through microinjection and identified by PCR and Southern blot. The following primers were used to identify the potentially GM sheep: cts, 5'-tacggtaaactgcccacttg-3' and cta, 5'-acctggagaagttatggctg-3'; tsf, 5'-gagccgtaaggtgattgtcgtg-3' and tsr, 5'-gcattcattttatgtttcaggttca-3'. The GM sheep were finally confirmed by Southern blot analysis (Roche Diagnostics, Mannheim, Germany). Probes were then generated by PCR using the following primers: Ps, 5'-actggtaaagaacttggaggaggg-3' and Pr, 5'-gtttcaggttcagggggaggtg-3'.

### Expression of *TLR4* in monocytes from GM and WT sheep

Sheep blood samples were collected from the jugular veins of the GM and WT sheep. A lymphocyte separation medium (TBD, Tianjin, China) was used to isolate monocytes and then total RNA was extracted using TRIzol method (Invitrogen, Carlsbad, CA, USA). Following the manufacturer’s instructions, cDNA was synthesized using a Thermo Scientific Revert Aid First Strand cDNA Synthesis Kit #K1611 (Thermo Fisher Scientific Inc., Vilnius, Lithuania). Real-Time PCR was used to assess the relative expression of the *TLR4*. *TLR4* and *β-actin*-specific primers were designed (*TLR4*: F, 5'-atcatcagcgtgtcggttgtca-3' and R, 5'-gcagccagcaagaagcatcag-3'; *β-actin*: F, 5'-caccgcaaatgcttctaggc-3' and R, 5'-ccatcccagcctcataaccc-3'). The qPCR reactions were set up manually and run on an Mx3000P instrument (Agilent Technologies, Santa Clara, CA, USA) and amplification data were analyzed using the Mx3000P software. The relative expression was determined using the comparative 2^−ΔΔCT^ method.

### Composition analysis of meat and rat diet

After removing the head, limbs, and entrails, as well as excessive fat from the sheep carcass, the remaining meat was minced and measured. Nutritional values (crude protein, fat, phosphorus, and ash) were analyzed in accordance with the standard methods (Chinese Standard GB/T5009.3–6, 10–2003). Based on the results of the compositional analysis, the rodent diets were supplemented with 3.75%, 7.5% or 15% (wt/wt) meat powder of GM and WT sheep, respectively, which met the required standard. Additionally, a meat-free commercial diet (CD) was used as the negative control. All diet formulations were irradiated with ^60^Co and were vacuum packed at Ke Ao Xie Li Feed Co. Ltd (Beijing, China).

### Rat housing

One hundred-forty weaning Sprague-Dawley rats with a body weight of 150–200 g and around 5 weeks of age were obtained from the Peking University Experimental Animal Center (Beijing, China). Animals were individually housed in a 12:12h light/dark cycle (lights on from 08:00 to 20:00) with food and water available *ad libitum*. The animal room temperature was maintained at 22±3°C, relative humidity was held at 30–70%, and air was exchanged 15 times/hour. The animals were acclimatized with the CD for 5 days before the study started, at which point they were randomly divided into 14 groups of either 10 male and 10 female rats with similar initial body weights.

### Observation and examination of rats

#### Clinical evaluation

Each rat was observed twice daily for mortality and signs of morbidity as well as for any other clinical signs of toxicity. Their body weights were measured weekly for the duration of the study.

### Hematology and blood biochemistry

On day 90 of the study, blood samples were obtained from the orbital sinus of the rats under anesthesia. Samples were collected for hematology and placed in tubes containing heparin. Samples collected for blood biochemistry were centrifuged, after which the supernatants were collected for evaluation.

White blood cell count (WBC), lymphocyte percentage (LY%), neutrophils percentage (NE%), monocyte percentage (MO%), red blood cell count (RBC), hemoglobin concentration (HB), hematocrit (HCT), mean corpuscular hemoglobin (MCH), blood platelet count (PLT), and mean platelet volume (MPV) were measured with a Sysmex F-820 blood cell counter (Sysmex Corporation, Kobe, Japan). The data recorded on the WBC (10^9^/L) and RBC (10^9^/L) were analyzed using lg (log_10_) transformations. Alanine aminotransferase (ALT), aspartate aminotransferase (AST), total protein (TP), albumin (ALB), alkaline phosphatase (ALP), glucose (GLU), blood urea nitrogen (BUN), creatinine (CREA), cholesterol (CHO), triglyceride (TG), lactate dehydrogenase (LDH), calcium (Ca), phosphorus (P), chlorine (Cl), and magnesium (Mg) were all measured with an RA-1000 auto-analyzer (Technicon, Tarrytown, NY, USA).

### Organ weight and histopathology

A complete gross necropsy was conducted by visual inspection on all of the rats at the end of the study. Selected organs were trimmed and relative weights (as a percentage of the final body weight) were determined for brain, heart, kidney, liver, lung, spleen, thymus, testes and ovaries. Paired organs were weighed together. Tissue sections from the brain, heart, liver, lung (with bronchi), spleen, kidney, thymus, stomach, intestine, testes, and ovaries of the rats that consumed CD, 15% WT diet, and 15% GM diet were fixed with 10% buffered formalin. Paraffin-embedded tissues were sectioned at a thickness of 2–5 μm and stained with hematoxylin and eosin. A histopathological examination of tissue sections was conducted at the Experimental Animal Research Center located at China Agricultural University (Beijing, China).

### Statistical analysis

Statistical comparisons were conducted to determine whether differences between the values of the aforementioned response variables among different treatment groups were attributable to the consumption of diets that were formulated with GM meat compared with WT meat. For each sex, combined group data variances were analyzed using Tukey’s HSD test. A one-way analysis of variance (ANOVA) was applied to evaluate the homogeneity variance. This process was followed by conducting a least squared differences model using SAS v9.0 (SAS Inc., Cary, NC, USA) to detect the different variables between the combined groups. The data were presented as mean values and standard deviation. Differences were considered significant when *p* < 0.05.

## Results

### Production and screening of GM sheep

**Fig 1:**
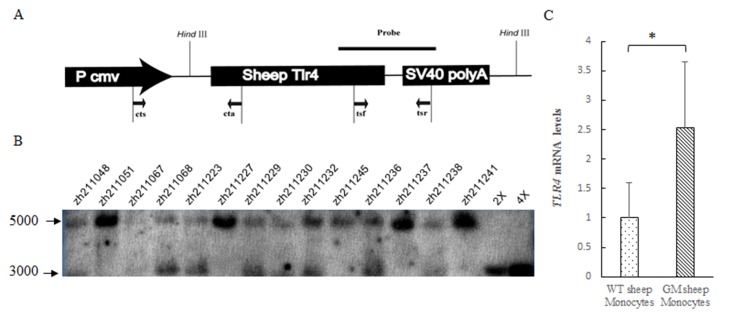
Generation and identification of GM sheep and analysis for *TLR4* expression in monocytes. (A) The expression vector. Sheep *TLR4* was inserted into the vector, which had the promoter CMV and the SV40 polyA. The cts, cta and tsf, tsr were used as PCR primers and DIG-labeling Probes were used to identify GM sheep. (B) Southern blot analysis of *HindIII*-digested sheep genomic DNA. Lane serial dilutions (2× and 4×) of transgene plasmid DNA spiked into genomic DNA of WT sheep. Lower arrow (about 3k bp) indicated the exogenous *TLR4*. The GM sheep were: zh211048, zh211067, zh211068, zh211223, zh211229, zh211230, zh211232, zh211236, zh211238 and the WT sheep were: zh211051, zh211227, zh211245, zh211237, zh211241. (C) The mRNA expression of *TLR4* in monocytes was quantified using RT-PCR. Asterisk (*) represents the significant difference between groups (*p* < 0.05). GM: genetically modified, WT: wild-type.

The linear fragment containing the sheep *TLR4* gene was microinjected into the pronuclei (Fig 1A). Afterwards, Founder sheep were bred with WT animals of the same strain, which then transmitted the gene construct to their offsprings. F1-generation sheep were identified by two pairs of primers, cts, cta, and tsf, tsr, as well as by Southern blot method (Fig 1B). The endogenous *TLR4* showed a band of 5 kb (the gene of *TLR4* was in chromosome 2, and after digesting the genome of the sheep using *HindIII*, the fragment that contained the gene *TLR4* was 4898 bp). The exogenous *TLR4* band was about 3000 bp. Furthermore, the mRNA expression of *TLR4* in the GM sheep monocyte was 2.5-fold compared with the WT sheep monocyte using qPCR (Fig 1C). These results indicated that GM sheep overexpressing *TLR4* were successfully obtained.

### Compositional analysis of meat and rodent diets

Water, crude protein, crude fat, vitamin (A, E, B1, B2) and amino acid concentrations of GM and WT sheep meat are shown in Table 1. The concentrations of water, protein and fat were similar to a previous report [19]. The rodent diet formulations included 3.75%, 7.5% or 15% (wt/wt) meat powder. All of the fiber, energy and amino acids met the nutrient specifications for rodents as per the diet request of GB 14924.3–2010 (Table 2).

**Table 1:** Compositional analyses of sheep meat.

	**GM sheep meat**	**WT sheep meat**
**Component (%)**		
Moisture	74.50	76.46
Protein	20.76	20.10
Fat	3.63	2.15
Phosphorus	0.16	0.17
Ash	1.05	0.92
**Vitamins (mg/100g)**		
A	0.0070	0.0144
E	0.0686	0.1020
B1	0.0276	0.0465
B2	0.0875	0.0823
**Amino acid compositions (g/100g)**		
Asp	0.50	0.49
Thr	0.40	0.33
Ser	0.42	0.61
Glu	1.60	1.50
Gly	1.40	0.82
Ala	0.71	0.52
Val	1.00	0.72
Met	0.50	0.28
Ile	0.91	0.66
Leu	1.80	1.40
Tyr	0.61	0.46
Phe	0.58	0.61
Lys	1.90	1.40
His	0.64	0.46
Arg	1.40	1.00

GM: genetically modified, WT: wild-type.

**Table 2:** Nutritional analysis of rodent diets.

	****CD****	****WT sheep meat****			****GM sheep meat****		
		****3.75%****	****7.5%****	****15%****	****3.75%****	****7.5%****	****15%****
**Item (g/100g)**							
Meat powder		3.75	7.50	15.00	3.75	7.50	15.00
Corn	28.70	44.65	42.00	39.00	45.25	43.80	38.00
Middling	20.00						
Flour	15.00						
Fish meal	1.50						
Soybean meal	23.00	36.00	34.60	30.60	36.00	34.00	30.00
Sugar beet pulp	4.50	5.00	6.00	6.00	5.00	6.00	9.90
Vegetable oil	1.60	4.40	3.70	2.20	3.80	2.50	0
Mountain flour	1.40	1.50	1.40	1.40	1.50	1.40	1.30
Beer yeast power	1.00	1.00	1.00	2.00	1.00	1.00	2.00
Calcium hydrogen phosphate	1.80	2.20	2.30	2.30	2.20	2.30	2.30
Salt	0.50	0.50	0.50	0.50	0.50	0.50	0.50
Additive 1%	1.00	1.00	1.00	1.00	1.00	1.00	1.00
**Nutrients (g/100g)**							
Dry matter	88.40	87.91	88.26	88.94	87.84	88.10	88.69
Crude protein	20.50	20.57	20.59	20.56	20.64	20.53	20.64
Crude fat	4.00	7.41	7.42	7.39	7.40	7.41	7.44
Crude fiber	2.80	3.85	3.93	3.65	3.86	3.92	4.39
Crude ash	6.30	3.07	3.02	2.84	3.08	3.01	2.95

CD: commercial diet, GM: genetically modified, WT: wild-type.

### Body weight

**Fig 2:**
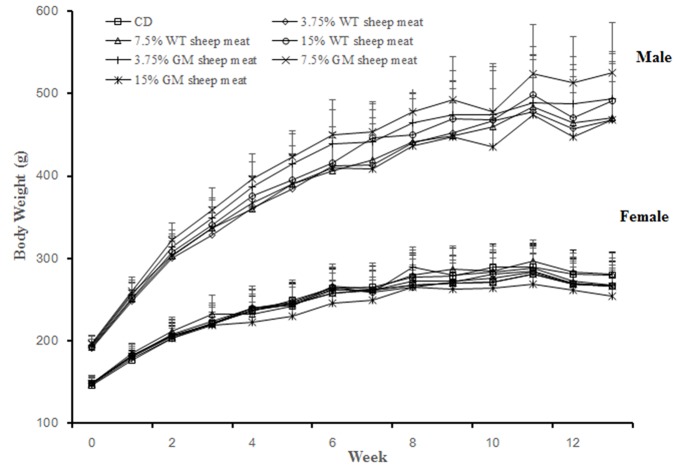
Mean live body weight of rats. CD: commercial diet, GM: genetically modified, WT: wild-type.

The mean weekly body weights for male and female rats in both the GM sheep meat groups and the WT sheep meat groups were similar, as shown in Fig 2. At week 7 and week 13, a slight reduction in body weight was noted and possibility due to the changes in the feeding period prior to blood collection and the additional stress caused by the blood sampling procedure. Therefore, it was not considered to be treatment related. These results were similar to results found in another study [20]. From week 10 to week 13, the body weights of rats were in a plateau phase, similar to results reported in previous studies [21, 22].

### Liver function

**Fig 3:**
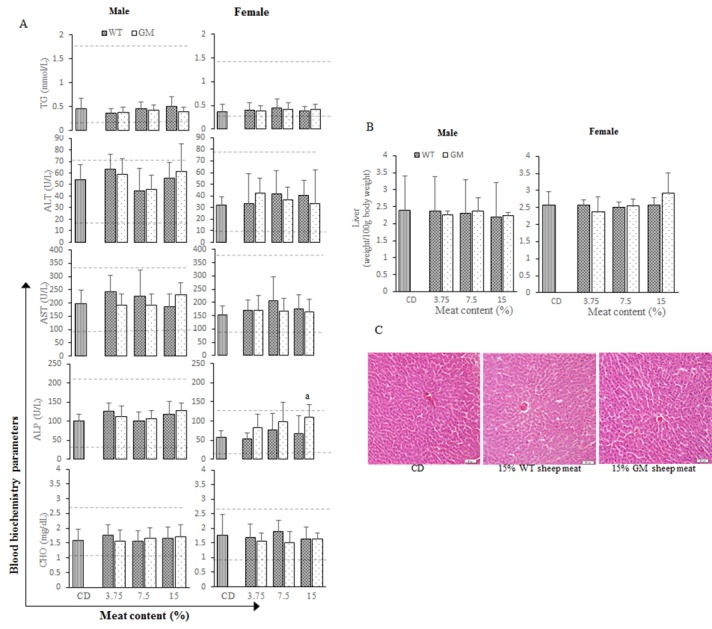
Hepatic function of male and female rats. (A) Blood biochemistry parameters of TG, ALT, AST, ALP, CHO for liver functions of rats fed with CD, 3.75% WT sheep meat, 3.75% GM sheep meat, 7.5% WT sheep meat, 7.5% GM sheep meat, 15% WT sheep meat, 15% GM sheep meat. The dashed lines represent the reference ranges. (B) Relative weight of livers from rats fed with CD, 3.75% WT sheep meat, 3.75% GM sheep meat, 7.5% WT sheep meat, 7.5% GM sheep meat, 15% WT sheep meat, 15% GM sheep meat. (C) Liver tissues from rats fed with CD, 15% WT sheep meat, 15% GM sheep meat. ^a^ Significant differences between meat and CD groups (*p* < 0.05). ^b^ Significant differences between GM group and corresponding WT group (*p* < 0.05). The reference ranges were obtained from the results of laboratory experiments with more than 1000 rats. CD: commercial diet, GM: genetically modified, WT: wild-type.

The most common values that served as markers of liver function were AST, ALT, CHO, ALP and TG, as shown in Fig 3A. Most of the findings were similar between the GM meat and WT meat diet groups.

The mean ALP value (109.80±30.96) observed in female rats that consumed 15% GM diet exceeded the value observed in the CD group (57.60±17.40). However, the mean ALP value of female rats consuming 15% GM was not significantly different from that of the 15% WT group (67.80±46.30). Additionally, the ALP value of the female rats that consumed the 15% GM diet was within the range of previous control rats (female, 12.4–130.8).

No significant difference was observed in the mean relative weight of the livers from rats among the various groups and from both sexes (Fig 3B). Additionally, the indexes of hepatic function such as TG, ALT, AST and CHO, were within the normal ranges in all of the animals in the GM group. Furthermore, as shown in Fig 3C, normal tissue architecture with no congestion were observed in the sinusoids of all groups. Occasionally, however, some fatty degeneration was observed in some hepatocytes. No gross pathological findings were observed during the necropsy and group-related histopathologic observations.

### Kidney function

**Fig 4:**
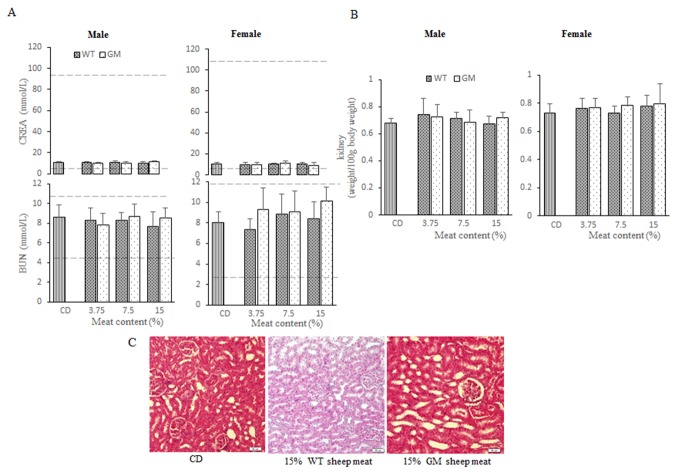
Kidney function of male and female rats. (A) Blood biochemistry parameters of BUN, CREA for kidney functions of rats fed with CD, 3.75% WT sheep meat, 3.75% GM sheep meat, 7.5% WT sheep meat, 7.5% GM sheep meat, 15% WT sheep meat, 15% GM sheep meat. The number of rats were 10 rats/sex/group. The dashed lines represent the reference ranges. (B) Relative weight of kidneys from rats fed with CD, 3.75% WT sheep meat, 3.75% GM sheep meat, 7.5% WT sheep meat, 7.5% GM sheep meat, 15% WT sheep meat, 15% GM sheep meat. (C) Kidney tissues from rats fed with CD, 15% WT sheep meat, 15% GM sheep meat. ^a^ Significant differences between meat and control groups (*p* < 0.05). ^b^ Significant differences between GM group and corresponding WT group (*p* < 0.05). The reference ranges were obtained from the results of laboratory experiments conducted with more than 1000 rats. CD: commercial diet, GM: genetically modified, WT: wild-type.

No significant differences were observed in BUN or CREA in either the male or female groups, which were the parameters that reflected kidney function (Fig 4A). Furthermore, there were no significant differences between the relative kidney weights among the groups and the sexes (Fig 4B). No gross pathological findings during necropsy or group-related histopathologic observations were found. Normal glomus body and renal capsule were observed. As shown in Fig 4C, some renal casts were occasionally observed. Adrenal histologic findings showed no significant difference in the thickness in the glomerular capillaries among the groups. These results suggested that the kidney functions were normal.

### Hematology and blood biochemistry

No differences were observed in most of the blood parameters between the GM and WT groups (Table 3). There were some differences, however, between the CD and WT groups, and between the CD and GM groups. The suspected reason for these differences was dependent upon whether or not the rats received a meat-based diet [23]. This research mainly discussed the differences in the parameters of the GM and WT meat diet groups. For the female rats, the mean MO% value (4.19±1.04) of the 3.75% GM group was lower than the 3.75% WT group (6.26±1.58). The mean values of WBC (9.80±0.21) and HCT (46.92±9.12) in the rats that consumed the 15% GM diet exceeded the corresponding group whose levels were 9.56±0.18 and 36.55±5.00, respectively. For the male rats, the mean value of MCH in rats that consumed the 7.5% GM diet (15.05±0.60) was below the mean value of MCH found in the 7.5% WT group (16.06±0.48).

**Table 3:** Blood parameters for female and male rats (mean±SD) (n = 10).

**Parameters**	**CD**	**WT sheep meat**			**GM sheep meat**			**Reference ranges**
		**3.75%**	**7.5%**	**15%**	**3.75%**	**7.5%**	**15%**	
**Female**								
WBC (lg10^9^/L)	9.74±0.19	9.64±0.10	9.59±0.19	9.56±0.18	9.68±0.15	9.72±0.16	9.80±0.21^b^	9.36–10.04
LY (%)	63.67±4.31	68.82±6.07	73.26±7.21^a^	69.06±7.42	71.34±6.23	72.54±7.74	67.83±8.58	—
NE (%)	27.66±3.98	23.10±5.71	19.18±5.63^a^	23.86±6.83	22.61±5.61^a^	21.44±6.98^a^	26.25±7.57	—
MO (%)	5.99±1.87	6.26±1.58	5.79±1.23	5.62±1.20	4.19±1.04^b^	4.89±1.47	4.77±1.29	—
RBC (lg10^12^/L)	12.83±0.06	12.84±0.06	12.84±0.06	12.83±0.06	12.86±0.09	12.86±0.11	12.93±0.08	12.52–13.02
PLT (10^9^/L)	727.60±157.15	839.00±124.16	714.70±135.34	724.50±91.09	710.00±129.69	707.20±107.05	776.00±201.73	240.18–1031.27
MPV (fl)	6.66±0.70	6.22±0.39	6.00±0.27^a^	6.11±0.48	6.08±0.30	6.29±0.26	6.75±0.67	4.52–7.24
HB (g/L)	122.40±17.45	120.20±17.05	113.80±17.15	116.80±15.53	123.60±30.13	130.30±40.76	150.80±29.73	39.67–200.83
HCT (%)	38.92±5.84	38.37±4.91	34.99±4.99	36.65±5.00	37.95±7.33	39.08±10.9	46.72±9.12^b^	19.44–65.48
MCH (fl)	17.87±0.93	17.24±1.09	16.39±0.75^a^	16.96±0.65	16.79±0.68	17.19±1.09	17.55±0.97	6.41–31.61
MCHC (pg)	315.00±10.69	313.00±13.07	324.90±13.92	319.00±10.01	323.50±18.82	331.50±13.89	322.80±18.26	8.08–561.52
Ca (mmol/L)	2.22±0.24	2.24±0.19	2.32±0.12	2.39±0.31	2.21±0.18	2.30±0.22	2.12±0.78	1.70–3.30
P (mg/dL)	2.41±1.60	1.92±0.45	2.77±0.72	2.32±0.85	2.23±0.63	2.42±0.81	2.20±1.11	1.50–3.90
Cl (mmol/L)	102.53±2.74	104.31±2.75	105.63±2.52	106.60±7.04	102.99±1.96	107.14±5.04	107.49±4.66	—
Mg (mmol/L)	1.10±0.29	1.06±0.09	1.17±0.14	1.10±0.12	1.04±0.08	1.05±0.15	1.11±0.13	—
**Male**								
WBC (lg10^9^/L)	9.95±0.14	9.83±0.14	9.69±0.21^a^	9.82±0.17	9.81±0.14	9.86±0.10	9.73±0.10^a^	9.33–9.95
LY (%)	65.21±8.07	62.12±5.29	61.25±8.92	61.72±9.60	65.47±6.43	68.28±9.39	52.06±8.82^a^	—
NE (%)	29.53±6.93	30.40±4.63	29.94±8.58	29.15±9.75	26.74±5.87	24.00±8.68	38.59±8.17	—
MO (%)	4.87±1.94	6.92±1.89	8.10±2.34 ^a^	7.85±2.00^a^	6.87±1.58	6.95±1.49	8.75±2.26^a^	—
RBC (lg10^12^/L)	12.94±0.02	12.90±0.04	12.86±0.06	12.89±0.05	12.87±0.09	12.86±0.08	12.93±0.04	12.47–12.98
PLT (10^9^/L)	749.17±151.57	661.5±105.59	484.3±300.26^a^	599.5±100.38	623.13±121.67	621.60±96.47	617.22±130.71	240.18–1031.27
MPV (fl)	5.08±0.35	5.98±0.58^a^	5.90±0.88 ^a^	5.58±0.45	5.55±0.43	5.34±0.32	5.57±0.64	4.12–7.64
HB (g/L)	141.3±12.24	128±15.53	116.5±15.23^a^	120.6±15.99	117±25.47^a^	111.6±21.19^a^	131.33±14.12	39.67–200.83
HCT (%)	44.43±3.06	41.17±3.62	36.98±5.14^a^	39.03±5.38	36.94±8.14^a^	35.51±6.51^a^	40.94±4.70	19.44–65.48
MCH (fl)	16.33±0.79	15.88±0.91	16.06±0.48	15.38±0.61^a^	15.29±0.75^a^	15.05±0.60^a^ ^,^ ^b^	15.47±0.50^a^	6.41–31.61
MCHC (pg)	317.80±10.36	310.20±13.68	315.40±12.17	309.30±14.17	317.00±12.65	313.90±8.31	321.10±13.30	8.08–561.52
Ca (mmol/L)	2.13±0.16	2.20±0.16	2.18±0.23	2.25±0.14	2.07±0.13	2.21±0.17	1.97±0.21^b^	1.51–3.19
P (mg/dL)	2.90±1.27	2.77±0.76	2.86±0.45	2.94±0.47	2.73±0.74	2.87±0.61	2.76±0.71	1.70–4.10
Cl (mmol/L)	102.90±1.38	105.90±3.08	106.27±2.44^a^	106.29±2.92^a^	103.19±2.24	104.85±1.91	106.67±2.78^a^	—
Mg (mmol/L)	1.10±0.17	1.08±0.11	1.03±0.07	1.03±0.08	1.03±0.14	1.08±0.08	1.06±0.10	—

^a^ Significant differences between meat and CD group *(p* < 0.05).

^b^ Significant differences between GM and corresponding WT group (*p* < 0.05).

The reference ranges were obtained from the results of previous laboratory experiments.

CD: commercial diet, GM: genetically modified, WT: wild-type.

### Organ weight and histopathology

The mean relative weights of organs in the GM meat groups were similar to those found in the WT meat groups. No differences were observed between the GM and WT groups (Table 4).

**Table 4:** Relative weight (weight/100 g body weight) ratio for female and male rats (mean±SD, %) (n = 10).

	CD	WT sheep meat			GM sheep meat		
		3.75%	7.5%	15%	3.75%	7.5%	15%
**Female**							
brain	0.678±0.063	0.697±0.084	0.697±0.049	0.712±0.109	0.707±0.050	0.689±0.082	0.727±0.065
spleen	0.193±0.033	0.192±0.050	0.172±0.031	0.188±0.038	0.161±0.016	0.178±0.043	0.181±0.026
heart	0.372±0.028	0.370±0.043	0.348±0.026	0.371±0.039	0.368±0.046	0.365±0.046	0.391±0.051
lung	0.519±0.112	0.574±0.175	0.592±0.105	0.591±0.092	0.611±0.113	0.580±0.128	0.574±0.090
thymus	0.121±0.027	0.129±0.032	0.110±0.018	0.123±0.022	0.109±0.019	0.106±0.033	0.118±0.026
adrenal gland	0.030±0.005	0.032±0.007	0.030±0.009	0.034±0.009	0.032±0.005	0.032±0.006	0.039±0.009^a^
ovaries	0.059±0.011	0.060±0.015	0.059±0.007	0.074±0.019^a^	0.062±0.013	0.063±0.012	0.066±0.012
**Male**							
brain	0.421±0.019	0.440±0.057	0.452±0.042	0.421±0.041	0.407±0.069	0.419±0.048	0.448±0.042
spleen	0.156±0.020	0.144±0.021	0.158±0.025	0.156±0.033	0.148±0.017	0.144±0.034	0.143±0.016
heart	0.350±0.034	0.348±0.057	0.342±0.032	0.362±0.021	0.323±0.031	0.322±0.051	0.359±0.029
lung	0.485±0.182	0.407±0.078	0.453±0.058	0.446±0.073	0.439±0.100	0.415±0.042	0.441±0.075
thymus	0.093±0.020	0.101±0.030	0.118±0.039	0.080±0.021	0.085±0.048	0.097±0.038	0.096±0.018
adrenal gland	0.015±0.003	0.014±0.003	0.015±0.002	0.014±0.002	0.014±0.003	0.023±0.033	0.014±0.004
testes	0.683±0.051	0.698±0.110	0.686±0.085	0.689±0.054	0.695±0.080	0.610±0.079	0.697±0.094

^a^ Significant differences between meat and CD groups (*p* < 0.05).

^b^ Significant differences between GM and corresponding WT group (*p* < 0.05).

CD: commercial diet, GM: genetically modified, WT: wild-type.

There were no remarkable gross pathological alterations in any of the animals during the necropsy, nor were there any alterations in the histopathological examination related to the different diets. In the digestive system, liver cells experienced occasional fatty degeneration, and mucosa and villus desquamation were observed in the duodenum and jejunum. In the spleen, splenic capsules casually thickened. However, these histopathological results appeared to be incidental, as the frequency and severity of pathological changes were similar in the 15% GM group compared with those of the corresponding WT group and CD group (Table 5). Normal physiological changes observed in this study were common in many rats of this species and age, so the differences could not be attributed to the test substance.

**Table 5:** Summary of microscopic pathology observations (10 rats of each sex per group).

	CD		15% WT sheep meat		15% GM sheep meat	
Observation	Male	Female	Male	Female	Male	Female
Liver						
Fatty degeneration	5	0	1	5	0	2
Spleen						
Splenic capsule thickening	6	8	3	3	3	7
Duodenum						
Mucosa desquamation	2	0	2	0	2	1
Villus desquamation	0	1	0	0	0	0
Jejunum						
Mucosa desquamation	1	3	4	0	2	0
Villus desquamation	0	0	0	0	1	2

CD: commercial diet, GM: genetically modified, WT: wild-type.

Additionally, in the reproductive system, the testes and epididymides were normal in size and the relative weights of the testes were not significantly different between the treatments and the negative control. In the ovaries, some undergrown follicles were found, this appeared to be normal for rats that were between 18 and 20 weeks old. No abnormalities were observed in the brain, heart, thymus, lung and stomach. As these were normal physiological changes, the injuries or diseases to these tissue sections could not be considered harmful or were not caused by consuming GM meat.

## Discussion

Animal diseases have many adverse effects on human health, including antibiotic misuse, zoonotic infection and food safety. GM animals provide valuable models for investigating disease progression and evaluating different approaches to control diseases. Transgenic techniques could be used to promote animal welfare and improve livestock production. However, the introduction of GM animals into the food chain may cause concerns regarding food safety [24, 25].

In recent years, studies on the safety of GM food have been conducted. Most of these studies, however, concern GM crops and rarely include findings regarding GM farm animal food. To date, as known to the authors, only one safety assessment experiment on GM cattle meat has been conducted [26].

In the earlier studies, the exogenous protein of GFP and Cry1Ab/Ac could be digested without producing an allergic reaction [27, 28] and the TLR4 protein could be transferred to the endosome through the pathway Triad3A, Rab7b, and degenerated in the cytolysosome [29]. The sheep TLR4 protein is regarded harmless, or nontoxic, to mammals, including humans. In our study, we chose the *TLR4* gene derived from sheep and produced GM sheep overexpressing *TLR4* under the control of the promoter CMV. The relative expression of *TLR4* was about 2.5-fold in GM sheep compared with WT sheep. This may offer a new method for breeding disease-resistant animals.

To assess the safety of GM sheep meat, a 90-day rat feeding study was conducted according to the principles of OECD guideline 408. The study also followed the guidance on the risk assessment of food and feed from GM animals and on animal health and welfare aspects [30]. There were no significant differences between the meat nutrient composition of GM sheep and WT sheep. As revealed through an analysis of the liver and kidney functions, only the value of ALP in the female 15% GM group was significantly different from the CD group. However, even in this case, there were no significant differences between the 15% GM group and the 15% WT group. This may be because of the different dietary treatments, as one group had an animal-product-based diet and the other had a plant-based diet. Similar results had been reported in previous studies [23]. Neither the relative weight nor the histological analysis concerning the livers between the various groups was different. Therefore, the difference of ALP values might be related to the diet but not to the GM sheep meat specifically.

Compared with rats which were fed with WT meat, some differences were observed in the blood parameters of rats which were fed with GM meat, including WBC, MO%, HCT, MCH and Ca. Despite these differences, however, all of the parameters in rats in the GM group were within the normal reference ranges. Additionally, there were no significant findings in the immune organs (thymus and spleen). Furthermore, these differences were not relevant to other clinical or pathological findings, so individual rats might have contributed to the difference and were thus considered insignificant [31] A deeper exploration of the reasons for biological variation will be of benefit to minimize the differences during future animal experiments.

In this study, three test dose levels and a negative control were used. The meat powder supplements were adjusted to the low level of 3.75%, the medium level of 7.5%, and the high level of 15%. The average daily consumption of the diet for an adult rat was 20 g of food. When a rat weighing 150 g was fed a diet supplemented with 15% meat, the GM meat consumed was about 3 g, which would be roughly equivalent to 195 g of meat for a 60-kg adult person as suggested by the formula for dose translation [32]. In China, 3.0 kg of mutton was consumed per capita in 2009, which meant that on average about 8 g was consumed per day. According to the figures released for 2009, 57.3 kg of meat, including pork, poultry, beef and mutton, was eaten per capita in 1 year, which amounted to about 157 g consumed per day [33]. As a result, the value of 195 g in the current study was higher than the normal Chinese human dietary intake (157 g).

During the 90-day feeding study, there were no obvious or significant changes in the clinical signs and body weights in the rats that were fed different dietary formulations. Additionally, the hematology and the blood biochemistry results were all in the normal ranges for both groups of rats that consumed the GM meat and the WT meat. The results of the histopathology showed that there were no significant differences observed in the heart, liver, spleen, kidney, thymus, stomach, intestine, ovaries, or testes of rats in different groups. Similar results were also reported in other feeding studies with a diet containing ingredients derived from GM organisms [20, 21 26, 34].

## Conclusion

The current 90-day toxicology study indicated that the meat of GM sheep overexpressing *TLR4* had no adverse or toxic effects on Sprague-Dawley rats in comparison with WT sheep meat.

## Supporting Information

S1 Dataset Blood parameters of rats. (XLS)

S2 Dataset Body weights of rats. (XLS)

S3 Dataset Hepatic and renal parameters of rats. (XLS)

S4 Dataset Organ weights of rats. (XLS)

S1 Table Compositional analysis of sheep meat. (DOCX)

## References

[1] Hammer RE, Pursel VG, Rexroad CE Jr, Wall RJ, Bolt DJ, Ebert KM, et al Production of transgenic rabbits, sheep and pigs by microinjection. Nature. 1985;315: 680–683. 3892305 10.1038/315680a0

[2] Murray JD, Nancarrow CD, Marshall JT, Hazelton IG, Ward KA. Production of transgenic merino sheep by microinjection of ovine metallothionein-ovine growth hormone fusion genes. Reprod Fertil Dev. 1989;1: 147–155. 2552507 10.1071/rd9890147

[3] Huang YJ, Huang Y, Baldassarre H, Wang B, Lazaris A, Leduc M, et al Recombinant human butyrylcholinesterase from milk of transgenic animals to protect against organophosphate poisoning. Proc Natl Acad Sci U S A. 2007;104: 13603–13608. 17660298 10.1073/pnas.0702756104 PMC1934339

[4] Yang X, Carter MG. Transgenic animal bioreactors: a new line of defense against chemical weapons? Proc Natl Acad Sci U S A. 2007;104: 13859–13860. 17715298 10.1073/pnas.0706163104 PMC1955779

[5] Lyall J, Irvine RM, Sherman A, McKinley TJ, Nunez A, Purdie A, et al Suppression of avian influenza transmission in genetically modified chickens. Science. 2011;331: 223–226. 10.1126/science.1198020 21233391

[6] Richt JA, Kasinathan P, Hamir AN, Castilla J, Sathiyaseelan T, Vargas F, et al Production of cattle lacking prion protein. Nat Biotechnol. 2007;25: 132–138. 17195841 10.1038/nbt1271 PMC2813193

[7] Wall RJ, Powell AM, Paape MJ, Kerr DE, Bannerman DD, Pursel VG, et al (2005) Genetically enhanced cows resist intramammary Staphylococcus aureus infection. Nat Biotechnol. 2005;23: 445–451. 15806099 10.1038/nbt1078

[8] Clements JE, Wall RJ, Narayan O, Hauer D, Schoborg R, Sheffer D, et al Development of transgenic sheep that express the visna virus envelope gene. Virology. 1994;200: 370–380. 8178428 10.1006/viro.1994.1201

[9] Lee JY, Ye J, Gao Z, Youn HS, Lee WH, Zhao L, et al Reciprocal modulation of Toll-like receptor-4 signaling pathways involving MyD88 and phosphatidylinositol 3-kinase/AKT by saturated and polyunsaturated fatty acids. J Biol Chem. 2003;278: 37041–37051. 12865424 10.1074/jbc.M305213200

[10] Hoshino K, Takeuchi O, Kawai T, Sanjo H, Ogawa T, Takeda Y, et al Cutting edge: Toll-like receptor 4 (TLR4)-deficient mice are hyporesponsive to lipopolysaccharide: evidence for TLR4 as the Lps gene product. J Immunol. 1999;162: 3749–3752. 10201887

[11] Lu YC, Yeh WC, Ohashi PS. LPS/TLR4 signal transduction pathway. Cytokine. 2008;42: 145–151. 10.1016/j.cyto.2008.01.006 18304834

[12] Chow JC, Young DW, Golenbock DT, Christ WJ, Gusovsky F. Toll-like receptor-4 mediates lipopolysaccharide-induced signal transduction. J Biol Chem. 1999;274: 10689–10692. 10196138 10.1074/jbc.274.16.10689

[13] Karin M, Delhase M. The I kappa B kinase (IKK) and NF-kappa B: key elements of proinflammatory signalling. Semin Immunol. 2000;12: 85–98. 10723801 10.1006/smim.2000.0210

[14] Bihl F, Salez L, Beaubier M, Torres D, Lariviere L, Laroche L, et al Overexpression of Toll-like receptor 4 amplifies the host response to lipopolysaccharide and provides a survival advantage in transgenic mice. J Immunol. 2003;170: 6141–6150. 12794144 10.4049/jimmunol.170.12.6141

[15] Deng S, Li G, Zhang J, Zhang X, Cui M, Guo Y, et al Transgenic cloned sheep overexpressing ovine toll-like receptor 4. Theriogenology. 2013;80: 50–57. 10.1016/j.theriogenology.2013.03.008 23623352

[16] Deng S, Wu Q, Yu K, Zhang Y, Yao Y, Li W, et al Changes in the relative inflammatory responses in sheep cells overexpressing of toll-like receptor 4 when stimulated with LPS. PLoS One. 2012;7: e47118 10.1371/journal.pone.0047118 23056598 PMC3464238

[17] European Commission. Commission Implementing Regulation (EU) No 503/2013 of 3 April 2013 on applications for authorisation of genetically modified food and feed in accordance with Regulation (EC) No 1829/2003 of the European Parliament and of the Council and amending Commission Regulations (EC) No 641/2004 and (EC) No 1981/2006. Official Journal of the European Union. 2013;L157: 1–48

[18] Huang J, Wang Q. Biotechnology policy and regulation in China IDS Working Paper. Biotechnology Policy Series 4. Brighton: Institute of Development Studies 2003 Institute of Development Studies website. Available: http://www.ids.ac.uk/files/Wp195.pdf. Accessed 8 February 2015.

[19] Williams P. The Nutritional Composition of Red Meat. Nutrition & Dietetics. 2007;64: 113–119.

[20] Schroder M, Poulsen M, Wilcks A, Kroghsbo S, Miller A, Frenzel T, et al A 90-day safety study of genetically modified rice expressing Cry1Ab protein (Bacillus thuringiensis toxin) in Wistar rats. Food Chem Toxicol. 2007;45: 339–349. 17050059 10.1016/j.fct.2006.09.001

[21] Tang X, Han F, Zhao K, Xu Y, Wu X, Wang J, et al A 90-day dietary toxicity study of genetically modified rice T1C-1 expressing Cry1C protein in Sprague Dawley rats. PLoS One. 2012;7: e52507 10.1371/journal.pone.0052507 23300690 PMC3531449

[22] Sengupta P. A scientific review of age determination for a laboratory rat: how old is it in comparison with human age? Biomed Int. 2012;2: 81–89.

[23] Swanson KS, Kuzmuk KN, Schook LB, Fahey GC Jr. Diet affects nutrient digestibility, hematology, and serum chemistry of senior and weanling dogs. J Anim Sci. 2004;82: 1713–1724. 15216999 10.2527/2004.8261713x

[24] Whitelaw C, Sang H. Disease-resistant genetically modified animals. Revue scientifique et technique-Office international des épizooties. 2005;24: 275–283. 16110895

[25] Muller M, Brem G. Transgenic strategies to increase disease resistance in livestock. Reprod Fertil Dev. 1994;6: 605–613. 7569040 10.1071/rd9940605

[26] Liu S, Li CX, Feng XL, Wang HL, Liu HB, Zhi Y, et al (2013) Safety assessment of meat from transgenic cattle by 90-day feeding study in rats. Food Chem Toxicol. 2013;57: 314–321. 10.1016/j.fct.2013.04.003 23583492

[27] Richards HA, Han CT, Hopkins RG, Failla ML, Ward WW, Stewart CN Jr. Safety assessment of recombinant green fluorescent protein orally administered to weaned rats. J Nutr. 2003;133: 1909–1912. 12771338 10.1093/jn/133.6.1909

[28] Xu W, Cao S, He X, Luo Y, Guo X, Yuan Y, et al Safety assessment of Cry1Ab/Ac fusion protein. Food Chem Toxicol. 2009;47: 1459–1465. 10.1016/j.fct.2009.03.029 19341779

[29] Wang Y, Chen T, Han C, He D, Liu H, An H, et al Lysosome-associated small Rab GTPase Rab7b negatively regulates TLR4 signaling in macrophages by promoting lysosomal degradation of TLR4. Blood. 2007;110: 962–971. 17395780 10.1182/blood-2007-01-066027

[30] EFSA Panel on Genetically Modified Organisms (GMO), EFSA Panel on Animal Health and Welfare (AHAW). Guidance on the risk assessment of food and feed from genetically modified animals and on animal health and welfare aspects. EFSA Journal. 2012;10(1):2501 [43 pp.]. 10.2903/j.efsa.2012.2501 European Food Safety Authority website. Available: http://www.efsa.europa.eu/en/efsajournal/pub/2501.htm. Accessed 6 February 2015.

[31] Kampfmann I, Bauer N, Johannes S, Moritz A. Differences in hematologic variables in rats of the same strain but different origin. Vet Clin Pathol. 2012;41: 228–234. 10.1111/j.1939-165X.2012.00427.x 22551195

[32] Reagan-Shaw S, Nihal M, Ahmad N. Dose translation from animal to human studies revisited. FASEB J. 2008;22: 659–661. 17942826 10.1096/fj.07-9574LSF

[33] Zhou G, Zhang W, Xu X. China's meat industry revolution: challenges and opportunities for the future. Meat Sci. 2012;92: 188–196. 10.1016/j.meatsci.2012.04.016 22608925

[34] Tang M, Xie T, Cheng W, Qian L, Yang S, Yang D, et al A 90-day safety study of genetically modified rice expressing rhIGF-1 protein in C57BL/6J rats. Transgenic Res. 2012;21: 499–510. 10.1007/s11248-011-9550-6 21910016

