# Supplementary material for: A 90-Day Toxicology Study of Meat from Genetically Modified Sheep Overexpressing TLR4 in Sprague-Dawley Rats
Source: PLoS One. 2015 Apr 13;10(4):e0121636. doi: 10.1371/journal.pone.0121636 (PMC4395235; doi:10.1371/journal.pone.0121636)
Supplement: S1 Table — (DOCX) [file pone.0121636.s005.docx]

**Suprevision & Testing Center for GMOs food safety, Ministry of Agriculture (Beijing) Report**

No.JY130008A

| WT meat Unit | Value | criterion of detection |
| --- | --- | --- |
| Vitamin A mg/100g | 0.0144 | GB/T 5009.82-2003 |
| Vitamin E mg/100g | 0.102 | GB/T 5009.82-2003 |
| Vitamin B1 mg/100g | 0.0465 | GB 5413.11-2010 |
| Vitamin B2 mg/100g | 0.0823 | GB 5413.12-2010 |
| **Animo Acid** |  |  |
| Asp　　 g/100g | 0.49 | GB/T 5009.124-2003 |
| Thr 　　　　 g/100g | 0.33 | GB/T 5009.124-2003 |
| Ser　　　　 g/100g | 0.61 | GB/T 5009.124-2003 |
| Glu　　　　 g/100g | 1.5 | GB/T 5009.124-2003 |
| Gly 　　　　 g/100g | 0.82 | GB/T 5009.124-2003 |
| Ala　　　　 g/100g | 0.52 | GB/T 5009.124-2003 |
| Val　　　　 g/100g | 0.72 | GB/T 5009.124-2003 |
| Met　　　　 g/100g | 0.28 | GB/T 5009.124-2003 |
| Ile　 g/100g | 0.66 | GB/T 5009.124-2003 |
| Leu　　 g/100g | 1.4 | GB/T 5009.124-2003 |
| Tyr　　 g/100g | 0.46 | GB/T 5009.124-2003 |
| Phe　 g/100g | 0.61 | GB/T 5009.124-2003 |
| Lys　 g/100g | 1.4 | GB/T 5009.124-2003 |
| His　 g/100g | 0.46 | GB/T 5009.124-2003 |
| Arg　　 g/100g | 1.0 | GB/T 5009.124-2003 |

**Suprevision & Testing Center for GMOs food safety, Ministry of Agriculture (Beijing) Report**

No.JY130009A

| GM meat Unit | Value | criterion of detection |
| --- | --- | --- |
| Vitamin A mg/100g | 0.00707 | GB/T 5009.82-2003 |
| Vitamin E mg/100g | 0.0686 | GB/T 5009.82-2003 |
| Vitamin B1 mg/100g | 0.0276 | GB 5413.11-2010 |
| Vitamin B2 mg/100g | 0.0875 | GB 5413.12-2010 |
| **Animo Acid** |  |  |
| Asp　　 g/100g | 0.50 | GB/T 5009.124-2003 |
| Thr 　　　　 g/100g | 0.40 | GB/T 5009.124-2003 |
| Ser　　　　 g/100g | 0.42 | GB/T 5009.124-2003 |
| Glu　　　　 g/100g | 1.6 | GB/T 5009.124-2003 |
| Gly 　　　　 g/100g | 1.4 | GB/T 5009.124-2003 |
| Ala　　　　 g/100g | 0.71 | GB/T 5009.124-2003 |
| Val　　　　 g/100g | 1.0 | GB/T 5009.124-2003 |
| Met　　　　 g/100g | 0.50 | GB/T 5009.124-2003 |
| Ile　 g/100g | 0.91 | GB/T 5009.124-2003 |
| Leu　　 g/100g | 1.8 | GB/T 5009.124-2003 |
| Tyr　　 g/100g | 0.61 | GB/T 5009.124-2003 |
| Phe　 g/100g | 0.58 | GB/T 5009.124-2003 |
| Lys　 g/100g | 1.9 | GB/T 5009.124-2003 |
| His　 g/100g | 0.64 | GB/T 5009.124-2003 |
| Arg　　 g/100g | 1.4 | GB/T 5009.124-2003 |
|  |  |  |

**Ke Ao Xie LI Feed Co. Led (Beijing, China)**

| Parameters | GM meat | WT meat |
| --- | --- | --- |
| Moisture (%) | 74.5 | 76.46 |
| Protein (%) | 20.76 | 20.1 |
| Fat (%) | 36.31 | 21.48 |
| P (%) | 0.16 | 0.17 |
| Ash (%) | 1.05 | 0.92 |
